# Prospective Roles of Tumor Necrosis Factor-Alpha (TNF-α) in COVID-19: Prognosis, Therapeutic and Management

**DOI:** 10.3390/ijms24076142

**Published:** 2023-03-24

**Authors:** Zarina Mohd Zawawi, Jeevanathan Kalyanasundram, Rozainanee Mohd Zain, Ravindran Thayan, Dayang Fredalina Basri, Wei Boon Yap

**Affiliations:** 1 Virology Unit, Infectious Disease Research Centre, Institute for Medical Research, National Institutes of Health, Ministry of Health, Bandar Setia Alam, Shah Alam 40170, Malaysia; zarina.zawawi@moh.gov.my (Z.M.Z.); jeevan@moh.gov.my (J.K.); rozainanee@moh.gov.my (R.M.Z.); ravin@moh.gov.my (R.T.); 2 Center for Toxicology & Health Risk Studies, Faculty of Health Sciences, Universiti Kebangsaan Malaysia, Jalan Raja Muda Abdul Aziz, Kuala Lumpur 50300, Malaysia; 3 Center for Diagnostic, Therapeutic and Investigative Studies, Faculty of Health Sciences, Universiti Kebangsaan Malaysia, Jalan Raja Muda Abdul Aziz, Kuala Lumpur 50300, Malaysia; dayang@ukm.edu.my

**Keywords:** cytokines, COVID-19, inflammation, SARS-CoV-2, TNF-α

## Abstract

The coronavirus disease 2019 (COVID-19) became a worldwide concern at the beginning of 2020 and has affected millions. Several previous studies revealed the impact of the imbalanced innate immune response on the progression of COVID-19 and its disease outcomes. High levels of proinflammatory cytokines such as tumor necrosis factor-alpha (TNF-α) and interleukins are produced readily by innate immune cells to fight Severe Acute Respiratory Syndrome-Coronavirus-2 (SARS-CoV-2) infections. Nonetheless, cytokine-mediated inflammatory events are also linked to detrimental lung injury and respiratory failure, which can result in deaths among COVID-19 patients. TNF-α is amongst the early cytokines produced to mediate proinflammatory responses and enhance immune cell infiltration in response to SARS-CoV-2 infections. In COVID-19, TNF-α-mediated inflammation can cause detrimental tissue damage and gradually promotes lung fibrosis, which later results in pneumonia, pulmonary edema, and acute respiratory distress syndrome. This review, therefore, aims to deliberate the immunomodulatory roles of TNF-α in promoting inflammation and its relation with COVID-19 morbidity and mortality. In addition, this review also proposes the potential of TNF-α as a biomarker for the prognosis of severe COVID-19 and its related complications and as a molecular target for anti-TNF-α therapy.

## 1. Introduction

The Severe Acute Respiratory Syndrome-Coronavirus-2 (SARS-CoV-2) is a new beta-coronavirus that causes coronavirus disease 2019 (COVID-19) [1,2,3]. Generally, the infection outcomes vary from person to person and can range from symptomless or mild symptoms to severe respiratory symptoms and failure of multiple vital organs [4,5,6]. One of the severe forms of COVID-19 is acute respiratory distress syndrome (ARDS), which has been reported to be closely associated with the host’s innate immune response [7,8].

ARDS was reported to occur in nearly 16% of hospitalized COVID-19 patients with severe pneumonia [9]. Several proinflammatory cytokines such as tumor necrosis factor-alpha (TNF-α), interleukin (IL)-6, IL-2, IL-7, and IL-10 are involved in the development of cytokine release syndrome (CRS), which then contributes to the high morbidity and mortality of COVID-19 including ARDS [10,11,12]. In view of the active involvement of proinflammatory cytokines in the progression of COVID-19, they have been proposed to be part of the molecular targets for diagnosing, treating, and preventing the severe forms of COVID-19 [13]. Therefore, understanding the underlying mechanisms of SARS-CoV-2-induced cytokine storm is particularly critical for patient management and the development of effective treatment regimens.

Upon invasion of viral contagions, tumor necrosis factor-alpha (TNF-α) produced by macrophages and monocytes is one of the early effectors that alert the host’s immunity about the dangers. By binding to the compatible TNF receptor (TNFR1), TNF-α can subsequently induce cellular apoptosis, modulate innate immune responses to limit the replication of the infectious agents, and promote the infiltration of macrophages, dendritic cells, natural killer cells, and neutrophils to the affected area to control and clear the infections [14]. TNFR1 is found in almost all types of cells. Therefore, it can exert various modulating effects on a broad range of cells [15]. TNF-α is also a powerful inducer of nuclear factor kappa B (NF-κB) that is responsible for the expression of multiple proinflammatory genes in the cell nucleus [16]. However, excessive TNF-α production over an extended period can backfire on the host [17]. In rheumatoid arthritis (RA) patients, continuous release of TNF-α in the joint space can sustain tissue inflammation and, thus, leads to bone and cartilage impairment [18]. The use of TNF-α inhibitors has proven beneficial for RA management [19]. In addition, TNF-α blockers are also used to treat glucose tolerance and insulin sensitivity impairment seen in Type-2 diabetes mellitus [20]. In terms of viral diseases such as hepatitis C virus (HCV) infections, anti-TNF-α agents used to prevent overreactive TNF-α-related biological activities are proven safe and beneficial in delaying HCV reactivation that may lead to fatal liver failure. The anti-TNF-α therapy aims to control the active role of TNF-α in promoting liver inflammation and fibrosis in HCV-infected patients [21]. In SARS-CoV infections, it is worth noting that TNF-α-induced inflammation can increase the pathogenesis and preassembly of infective virus particles [22]; therefore, TNF-α inhibitors are recommended to reduce severe disease outcomes.

Hussell et al. (2001) suggested using TNF-α antagonists as potential therapeutics in viral diseases [23]. They discovered that the anti-TNF-α antibody treatment in mice challenged with respiratory syncytial and influenza viruses decreased the TNF-α level, reduced inflammatory cell infiltration into the lungs, lowered cytokine synthesis by T cells, and prevented severe illnesses without impairing the virus clearance. A recent study [24] demonstrated that patients taking anti-TNF-α drugs to improve immune-mediated inflammatory disease (IMID) conditions did not show compromised health after contracting COVID-19; on the contrary, they fared better than their counterparts. Considering the evidence, TNF-α can be a potential treatment target for COVID-19 caused by imbalanced immune responses, including cytokine storms observed in many severely ill COVID-19 patients. To better understand the underlying mechanism of action of TNF-α in modulating the host immune response, particularly in response to SARS-CoV-2 infections, this review aims to deliberate the association of TNF-α with the severity of COVID-19 and the possibility of employing TNF-α as a therapeutic target for improving severe COVID-19.

## 2. Inflammation in SARS-CoV-2 Infection

### 2.1. Activation of Innate Immune Response by SARS-CoV-2

**Figure 1:**
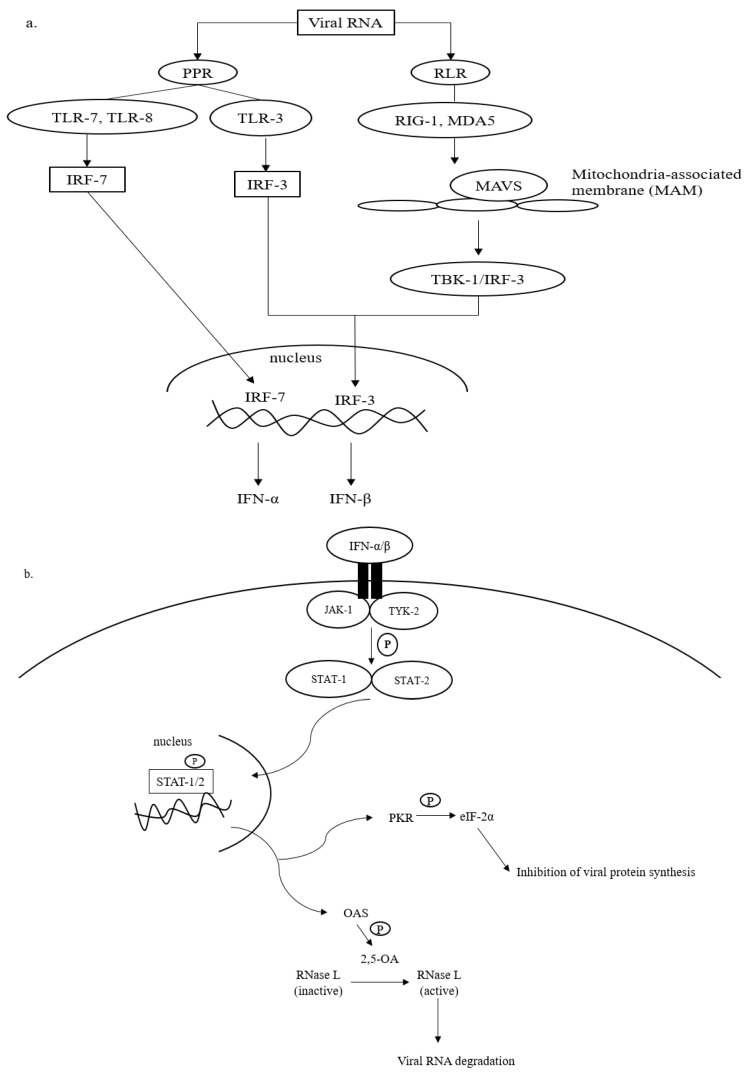
Antiviral defense of IFN. (**a**) Viral RNA (vRNA) can be recognized by pathogen pattern receptors (PPR) on the cell surface and retinoic acid-like receptors (RLR) in the cytosol. The vRNA sensing by PPR can be initiated by toll-like receptors (TLR)-3, -7, and -8, which then activates gene transcription factors, i.e., interferon regulatory factors (IRF)-3 and -7. IRF-3 and -7 are then translocated into the cell nucleus to express interferon (IFN)-β and interferon-α, respectively. The cytosolic viral dsRNA, on the other hand, is recognized by retinoic-like receptors (RLR) such as retinoic acid-inducible gene-1 (RIG-I) and melanoma differentiation-associated protein 5 (MDA-5). The RLR/vRNA complex subsequently binds to the mitochondrial-antiviral signaling (MAVS) protein located on the mitochondrial outer membrane. Upon the activation of MAVS, IRF-3 is phosphorylated via TANK-binding kinase (TBK)-1 to enhance the expression of IFN-β. (**b**) The antiviral cascades of IFN α/β demand the activation of Janus kinase/tyrosine kinase/signal transducer and activator of transcription (JAK/TYK/STAT) signaling. Activating the JAK/TYK/STAT transcription signaling pathway results in the expression of antiviral proteins, oligoadenylate synthetase (OAS), and protein kinase R (PKR), which are responsible for viral RNA degradation and inhibition of viral protein synthesis, respectively.

Upon SARS-CoV-2 infections, the innate immune response is armed, which is then actively involved in synthesizing proinflammatory cytokines, such as interferons (IFN) and chemokines [25,26]. The IFN-mediated antiviral defense is summarized in Figure 1.

Recognition of intruding viral pathogens by innate immune cells can be initiated via PPRs located on the cell surface, such as Toll-like receptor (TLR)-3, TLR-7, and TLR-8 [27,28]. TLR-3 recognizes viral double-stranded (ds) RNA during the initial phase of infections, whereas TLR-7 and TLR-8 that are found abundantly on bronchial epithelial cells and alveolar macrophages sense the presence of viral single-stranded (ss) RNA prior to the activation of the innate immune cascades (Figure 1a). Sensing vRNA by PPRs leads to the activation of gene transcription factors, namely interferon-regulatory factors (IRF)-3 and -7. IRF-3 and -7 are translocated into the cell nucleus and bind to their complementary promoters to induce the transcription of genes responsible for the synthesis of IFN-α and IFN-β.

In addition to PPRs, vRNA, especially dsRNA, can be detected by retinoic acid-like receptors (RLRs) in the cytosol (Figure 1a). RLRs that are often involved in the recognition of viral dsRNA include retinoid inducible gene 1 (RIG-I) and melanoma differentiation-associated gene 5 (MDA-5). The binding of dsRNA to RIG-1 or MDA-5 complexes that later interact with mitochondria-antiviral signaling (MAVS) protein on the outer membrane of mitochondria. The interaction promotes the phosphorylation of IRF-3 by TANK-binding kinase (TBK). As mentioned earlier, IRF-3 is translocated into the cell nucleus to initiate mainly the expression of IFN-β [27].

To amplify the antiviral response, the expressed IFN-α and -β bind to the compatible receptors, which subsequently initiates the phosphorylation of Janus (JAK) and tyrosine (TYK) kinases (Figure 1b). This leads to the activation of signal transducer and activator of transcription (STAT), particularly STAT-1 and -2. The activated STAT-1 and -2 are important transducers for expressing interferon-stimulated genes (ISGs) in the infected host cell [29]. The ISGs are then translated into antiviral molecules of innate immune response that actively battle virus infections, including oligoadenylate synthetase (OAS) and protein kinase R (PKR). OAS mediates the formation of 2,5-oligoadenylate (2,5-A) via ATP. In the presence of 2-5A, dimerization and activation of RNase L occurs in the cytosol, and the activated RNase L degrades vRNA to prevent viral genome replication and transcription (Figure 1b). PKR is responsible for the phosphorylation of eukaryotic initiation factor (eIF)-2α. The activated eIF-2α terminates viral protein translation [30]. In addition to the direct virucidal effects, the activation of OAS/RNAse L and/or PKR/eIF-2α mechanisms also promotes cellular stress, inflammation, and apoptosis, which in turn causes cell death. Collectively, both antiviral actions prevent further virus replication and spreading in the host [31].

Nonetheless, the perpetual virus infection increases the magnitude of host inflammatory events, resulting in more severe disease outcomes such as cytokine storms, vascular disruption, tissue damage, and organ failure [32]. In addition, to evade the host antiviral defense, viruses have evolved to form counteracting proteins that can interfere with the PPR recognition or the activation of innate immunity. Such a phenomenon is also observed in SARS-CoV-2 infections. The virus produces various structural and nonstructural proteins that can hijack the host’s innate response, suppressing type-I IFN production [33,34]. The inhibition can later affect the expression of antiviral molecules, such as PKR, that are responsible for downregulating the expression of essential viral proteins. Coronaviruses also interrupt the phosphorylation of eIF-2α to further impede the PKR-mediated antiviral response, especially in controlling the cellular translation machinery [35]. For example, the MERS-CoV Open Reading Frame (ORF)4a protein inhibits the binding of PKR to the viral dsRNA. The inhibition reduces the sensitivity of PKR in response to viral dsRNA and thus prevents the translational inhibition of viral proteins [36]. In regards to the inhibition of eukaryotic initiation factors, Xiao et al. (2008) found that at the later stages of SARS-CoV-1 infection, the viral S protein interacted with eIF-3F and subsequently permitted the overexpression of IL-6 and IL-8 that are known to promote an inflammatory response in acute virus infections [37]. Of note, the aforementioned modulations on the host antiviral response initially aim to sustain virus infections, including the novel SARS-CoV-2. However, the resultant increase of virus progeny invariably intensifies the activation of the host’s innate immunity and inflammatory response that eventually leads to hyperinflammation and severe disease complications in patients.

Besides inhibiting the biological activities of IFNs, SARS-CoV-2 also prevents cellular apoptosis to avoid elimination by the host immunity and concomitantly buy more time and space for its replication. Amongst the viral antigens of SARS-CoV-2, ORF3a shows anti-apoptotic effects by lowering the expression levels of apoptotic factors in host cells [38]. In another study that investigated the link between SARS-CoV-2 ORF3a and COVID-19 mortality among 20,000 patients from 23 different countries, the results indicated that ORF3a proteins of the virus isolates derived from countries with high infections and mortality cases were actively mutated [39]. The mutations explained the relatively high virus pathogenesis, disease morbidity, and mortality in those countries. Therefore, in conjunction with the other inhibitory actions on the host immunity, the virus can sustain its replication in hosts and cause the occurrence of severe COVID-19.

Despite the crucial roles of IFN and antiviral molecules in fighting virus infections, they often induce systemic inflammation that is highly possible to result in severe disease outcomes [40]. Plus, to date, several lines of evidence have linked the progression of severe forms of COVID-19 to CRS or cytokine storm syndrome (CSS) caused by the overproduction of proinflammatory cytokines and uncontrollable systemic inflammation [12,41,42]. For instance, Chen et al. (2021) reported that COVID-19 casualties exhibited greater levels of proinflammatory interleukins (IL-2, -6, -8, and -10) and TNF-α compared to the COVID-19 survivors [43]. A similar observation was also documented by Udomsinprasert et al. (2021), in which the circulating and locally produced IL-6, IL-10, and TNF-α were significantly increased in deceased COVID-19 patients [44]. These data imply that proinflammatory cytokines are among the main contributing factors causing severe forms of COVID-19, which are often linked to vital organ failure and death among patients [45].

Considering the more pronounced production of proinflammatory cytokines in deceased COVID-19 patients than in survivors, proinflammatory cytokines are deduced as a strong and independent predictor of patient survival [46,47]. Among the proinflammatory cytokines, the level of TNF-α is consistently higher in severe COVID-19 patients and casualties. Mortaz et al. (2021) investigated the serum level of soluble TNF-α receptors in healthy and COVID-19 patients [48]. The results indicated that the levels of TNF-α receptors were significantly higher in ICU and non-ICU COVID-19 patients than in healthy subjects. The observations reflect the feasibility of using TNF-α as a promising molecular predictor of severe COVID-19 in hospitalized patients.

The exact underlying impacts of SARS-CoV-2-induced hyperinflammation on severe COVID-19 patients are yet to be fully understood. However, the relatively high level of TNF-α in patients at the time of admission is strongly believed to be a contributing factor to irreversible organ damage and life-threatening complications [47,49]. Regardless of the active role of TNF-α in promoting the host’s innate immunity, a few reports found less significant implications of TNF-α in the morbidity and mortality of COVID-19 [50]. As a result, it remains relevant to further define, justify, and delineate the importance of TNF-α in the morbidity and mortality of COVID-19 due to the mixed and inconclusive clinical observations and laboratory analyses.

### 2.2. Activation of TNF-α Signaling Pathway by SARS-CoV-2 and Its Potential in Virus Containment

**Figure 2:**
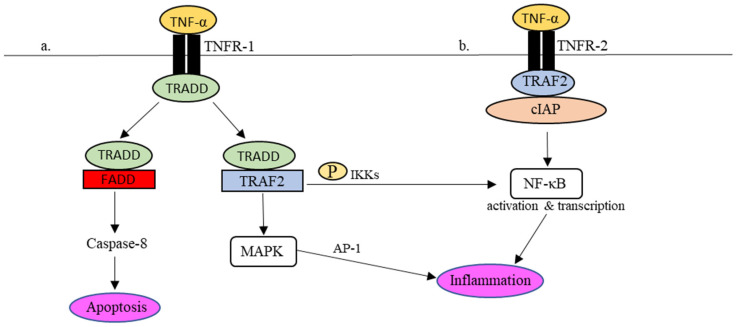
TNFR-1- and TNFR-2-mediated inflammatory responses in the TNF-α signaling pathway. (**a**) Upon TNFR-1 activation, TRADD is attracted to TNFR1 together with TRAF2 to phosphorylate NF-κβ and activate MAPK as well to regulate the expression of proinflammatory molecules. In addition, TRADD also binds to FADD, which leads to cell apoptosis via caspase-8. (**b**) In the event of TNFR-2 activation, the recruitment of TRAF2-cIAP complex to TNFR-2 activates the NF-κB pathway, which results in the regulation of inflammation.

Practically, in the presence of dangers, for instance, viruses, the production of TNF-α occurs almost instantly upon stimulation via PPRs [51,52]. TNF-α signaling is mediated by two cellular receptors, namely TNF receptor (TNFR)-1 and TNFR-2. Through interactions with TNFR-1 and -2, several intracellular antiviral cascades are awakened in response to the invading infectious agents [52,53]. Interactions between TNF-α and its receptors are summarised in Figure 2.

Upon binding to TNFR-1 (Figure 2a), the TNFR-1-associated death domain (TRADD) is attracted to the intracellular domain of TNFR-1. The recruitment of TRADD to TNFR-1 further attracts TNF receptors-associated factor 2 (TRAF-2) or Fas-associated protein with death domain (FADD) to control the downstream signaling actions. To activate the transcriptional gene factor, i.e., nuclear factor-kappa beta (NF-κβ), TRAF2 promotes the phosphorylation of NF-κβ by IκB kinases (IKKs). The phosphorylated NF-κβ is active and ready to perform proinflammatory gene transcription in the cell nucleus. In addition, the interaction between TRADD and TRAF-2 also encourages the activation of mitogen-activated protein kinase (MAPK), which in turn stimulates another transcriptional factor, known as activator protein-1 (AP-1), to regulate the expression of proinflammatory molecules, including cytokines and chemokines. As described earlier, uncontrollable cytokine production in response to viral infections may cause acute inflammation [54,55,56]. For example, NF-κβ-mediated IL-6 synthesis in COVID-19 was speculated to be associated with the adversity of the disease [57].

Synthesis of IL-6 through TNF-α/TNFR interactions was clearly demonstrated in rat C6 glioma cells [58]. The study outcomes emphasized the active involvement of MAPK and IκB-NFκB pathway in the expression of the IL-6 gene that later led to inflammation. The same observation was also reported by Tanabe et al. (2010) using a glioma cell line [59]. It further authenticated the role of TNF-α in promoting the production of IL-6 through the phosphorylation of NF-κB at serine residues located at positions 536 and 468. Accumulatively, it is agreeable that TNF-α has an active regulatory role in the early phase of innate immune response; its presence signifies the activation of innate immune response that subsequently leads to the synthesis of proinflammatory cytokines. As a result, the overproduction of proinflammatory cytokines during a cytokine storm seen in COVID-19 patients is unequivocally associated with the activation of the TNF-α signaling pathway.

The TRADD/FADD interaction (Figure 2a), on the other hand, activates the procaspase-8/caspase-3 apoptotic pathway. By undergoing cell apoptosis, the virus infection is contained, and the spreading of virus particles to the surrounding is limited. In short, TNFR-1 signaling can promote inflammation through NF-κB and MAPK signaling pathways and simultaneously causes cell apoptosis by activating caspase-8 [60,61]. Even though caspase-mediated apoptosis helps eliminated virally infected cells, it is worth noting that viruses such as SARS-CoV-2 have evolved multiple counter mechanisms to ensure survivability. Among others, SARS-CoV-2 ORF3a can alter caspase-8 and its activities and therefore lowers cell apoptosis and virus clearance [62].

TNFR-2, on the other hand, promotes intracellular signaling without inducing apoptosis, but it may assist in TNFR-I-dependent cell death [52]. It is noteworthy that TNFR-2 lacks a death domain. Contrary to TNFR-1, TNFR-2 directly interacts with the TRAF-2/cellular inhibitor of apoptosis (cIAP) complex (Figure 2b). The interaction then promotes the phosphorylation of NF-κB to initiate canonical and non-canonical NF-κB pathways for proinflammatory gene transcription. The hyperstimulation of NF-κB in patients with SARS and MERS occurs through the myeloid differentiation primary response 88 (MyD88) pathway and PPRs. It causes an intense proinflammatory response contributing to serious disease outcomes [63]. Given that a similar proinflammatory response is also observed in COVID-19, the severe forms of COVID-19 are most likely attributable to the hyperactivation of NF-κβ that regulates the synthesis and release of proinflammatory cytokines, such as TNF-α and IL-6, in severe COVID-19 patients. Interestingly, TNF-α can mediate the activation of the non-canonical NF-κB pathway, in which the proinflammatory response is more long-lasting regarding the production of inflammatory mediators [64]. The prolonged inflammation can therefore explain the more damaging cytokine storm and more serious respiratory distress and complications seen in COVID-19.

TNF-α is essential for protecting hosts from acute virus infections [65]. However, as a countermeasure, viruses can also dysregulate the functions of TNF-α to minimize viral clearance by TNF-α-mediated immune response. Using the zebrafish model, it was shown that viruses could impair TNF-α-induced viral clearance by resorting to the host autophagy response [66].

Considering the multifaceted role of TNF-α and various potential immune-escape strategies developed by viruses, it is of utmost importance to precisely define the functions of TNF-α as one of the key innate immune players in COVID-19 and SARS-CoV-2 infection so that it can be used as an independent COVID-19 biomarker. The information allows more systematic and efficient patient care and therapy.

## 3. Implication of TNF-α Signaling in COVID-19 Mortality and Morbidity

Although TNF-α signaling is critically required for activating innate immunity against infectious agents, dysregulation of TNF-α signaling can lead to severe complications, such as cytokine storm, that is importantly associated with deaths in COVID-19 patients. Regarding COVID-19-related lung injuries, post-mortem reports revealed bilateral widespread alveolar destruction and pulmonary edema with hyaline membrane development [67,68]. In view of the high levels of proinflammatory cytokines, particularly TNF-α in severe COVID-19 patients [69], it is speculated that the tissue destruction and pulmonary edema are most likely attributed to cytokine-regulated inflammation that causes the disintegration of endothelial and epithelial cytoskeletons, which subsequently enables influx of fluid during vasodilation [70].

In addition, TNF-α is also linked to bronchial hyperresponsiveness in COVID-19 patients, which causes them to suffer from decreased airway caliber and increased neutrophilia in respiratory epithelia. The occurrence of bronchial hyperresponsiveness indicates excessive TNF-α-mediated inflammation in the respiratory tract. As a result, the respiratory epithelia are exposed to aggressive inflammatory cytokine reactions, such as granulocyte-macrophage colony-stimulating factor (GM-CSF), IL-8, and intercellular adhesion molecules (ICAMs) [71]. Furthermore, to limit the replication and spread of SARS-CoV-2 in COVID-19 patients, TNF-α also promotes the influx of neutrophils to the respiratory tract. The recruited neutrophils are sensitized by the virus and its antigens and release matrix metalloproteinase (MMP)-9, which is strongly associated with irreversible pulmonary fibrosis in COVID-19 patients [72].

Overexpression of TNF-α causes more adverse complications in COVID-19 patients with comorbidities such as hypertension, obesity, and cardiovascular diseases (CVD). Several studies found that obesity was a critical predictor of severe COVID-19 [73]. Meanwhile, obese COVID-19 patients were reported to be 3.6 times more likely to end up in ICU wards [74]. In addition, based on a comprehensive data analysis of COVID-19 patients in China, Wu et al. (2020) found that patients with pre-existing cardiovascular complications had a mortality rate five times higher than those without CVD (10.5% versus 2.3%) [75]. The development of myocarditis in COVID-19 not only involves inflammatory cytotoxicity but also the dysfunction of receptors and ion channels due to the cross-reactivity of autoantibodies [76]. Those co-morbid individuals produce significantly higher levels of TNF-α that promote inflammation, endothelial dysfunction, and the development of atherosclerosis [77]. The inflammatory events result in altered coagulation propensity and blood clotting in the veins [78,79,80]. Chronically, it later contributes to the destabilization of atherosclerotic plaques and increases the risk of heart attack [81]. The condition is worsened by the overexpression of adhesion molecules that enable the adherence of leukocytes to endothelial. The activated leukocytes migrate into the lungs and cause localized inflammation, leading to vasodilation and edema [82].

After recovering from COVID-19, approximately 10–30% of non-hospitalized and 50–70% of hospitalized cases are linked to long COVID. Long COVID is a post-acute sequela of SARS-CoV-2 infection (PASC). It is manifested through a range of symptoms such as dyspnea, chest pain, memory loss, and pulmonary embolism that can persist for many months, even after the virus clearance [83]. While the exact underlying mechanism of long COVID is not fully understood, it is suggested that PASC individuals develop higher levels of TNF-α and IFN-γ–induced protein 10 (IP-10) than their counterparts during the early recovery phase [84]. Schultheiß et al. (2022) further observed persistently high levels of IL-1β, IL-6, and TNF-α in the plasma of individuals with PASC [85]. Therefore, the impact of TNF-α on long COVID and its mechanism of action should be further explored in order to deduce its adverse long-term complications to various body systems so that appropriate treatments and rehabilitation care can be provided accordingly to ease patients’ predicaments.

Collectively, because of the relationship between COVID-19, long COVID, and TNF-α, it highlights the significance of monitoring the TNF-α level in COVID-19 and PASC patients. It also bolsters the ideation of utilizing TNF-α inhibitors as potential therapeutic agents for the affected individuals.

## 4. Potential and Prospective Applications of TNF-α in COVID-19 Management

Severe COVID-19 is closely associated with cytokine storms caused by proinflammatory cytokines, such as TNF-α. It is, therefore, speculated that anti-inflammatory interventions targeting TNF-α can benefit COVID-19 patients, especially in preventing irreversible lung injury [13]. Amongst the proinflammatory cytokines found in COVID-19 patients and casualties, the expression of TNF-α and IL-6 outdoes that of their counterparts. Therefore, it is probable they can be developed into promising biomarkers for predicting severe forms of COVID-19 [47,86,87]. On top of that, comparing TNF-α and IL-6, the level of TNF-α has been consistently higher in severe COVID-19 patients and those who suffer from comorbidities such as obesity, hypertension, and chronic heart failure [73,74]. This provides robust evidence for adopting TNF-α as a more promising predictor for the prognosis of severe and life-threatening forms of COVID-19.

Additionally, the use of TNF-α as a protein biomarker has been reported in several disease diagnoses, such as cancers, Alzheimer’s disease, rheumatoid arthritis, and diabetes-related eye diseases [88]. In RA, high levels of TNF-α are found in the synovial fluid and patients’ sera, and the elevated expression of TNF-α correlates with the disease severity. It was found that an increase of one unit of the serum TNF-α activity could lead to a 9.6% rise in the likelihood of encountering severe RA [89]. As a result, it further implies the importance and feasibility of employing TNF-α in the prognosis of severe COVID-19 [90]. Ultimately, this approach not only benefits microbiology clinicians in making quick prognoses but also allows them to provide a more in-time treatment to COVID-19 patients.

Besides as a biomarker for the prognosis of severe COVID-19, targeting TNF-α using antibodies or inhibitors might reduce lung inflammation, hence a lower incidence rate of severe COVID-19 and death [91]. Izadi et al. (2021) explored the association of TNF-α monotherapy with the risk of hospitalization and death among adult patients with immune-mediated inflammatory diseases (IMIDs) [92]. In the study, the following treatment regimens were applied: (i) TNF-α inhibitor monotherapy; (ii) TNF-α inhibitor plus immunosuppressive methotrexate; (iii) TNF-α inhibitor plus immunosuppressive azathioprine/6-mercaptopurine; (iv) methotrexate monotherapy; (v) azathioprine/6-mercaptopurine monotherapy; and (vi) Janus kinase inhibitor monotherapy. The research team found that lower hospitalization and mortality were seen more in patients who received TNF-α inhibitor monotherapy than in the rest. Of note, the findings bolster the suggestion of adopting TNF-α inhibitors in reducing hospitalization and death rate among COVID-19 patients.

Salesi et al. (2021) investigated the occurrence of COVID-19 in 254 rheumatoid arthritis or seronegative spondyloarthropathy patients who were prescribed TNF-α blockers prior to the contraction of COVID-19 [93]. The study revealed that TNF-α blocker prescriptions like adalimumab, infliximab, and etanercept significantly decreased the risk of developing severe COVID-19 in the patients, with adalimumab having the highest preventive rate (96.8%) against severe COVID-19. Furthermore, rheumatoid arthritis patients taking anti-TNF medications were shown to have a lower hospitalization rate even if they caught COVID-19 [94]. Altogether, those reports support the benefits of TNF-α inhibitors in slowing the progression of COVID-19 and the potential of anti-TNF-α therapy in preventing adverse outcomes among COVID-19 patients.

Despite the complex and unclear pathogenesis of PACS, the absence of effective treatments could mean long-term and chronic health deterioration among long COVID patients; since previous findings suggested the involvement of TNF-α-induced inflammation in PASC and the manifestation of long COVID symptoms [84]. TNF-α inhibitors can help reduce complications experienced by long COVID individuals. Hypothetically, using TNF blockers for long COVID aims to reduce the intensity and severity of chronic inflammation that occurs due to SARS-CoV-2 infection, which could help alleviate some of the persistent symptoms. Therefore, employing anti-TNF-α agents can be included in the treatment regimen that deals with the imbalance of immune response in COVID-19 and PACS.

## 5. Concluding Remarks

The overexpression of TNF-α has been reported as an independent risk factor of death among COVID-19 patients with critical conditions. In conjunction with the other proinflammatory cytokines, TNF-α can induce localized and systemic inflammatory events that eventually lead to tremendous lung damage, pulmonary edema, and even death. This demonstrates the importance of TNF-α in the progression of COVID-19. Considering the profound immunomodulatory roles of TNF-α, it can potentially serve as a diagnostic or prognostic marker for severe COVID-19 and as a molecular target for TNF-α inhibitors in COVID-19 treatment and management. In the prognosis of COVID-19, changes in the level of TNF-α in comparison to the baseline can be used to predict the disease progression and severity, whereas using TNF-α inhibitors or blockers to treat COVID-19 can prevent mortality in severe COVID-19 patients. Conclusively, to precisely propose the use of TNF-α in the prognosis and treatment of COVID-19, further studies and rigorous empirical investigation are warranted to help people affected by COVID-19 in the near future.

## Data Availability

Not applicable as no new data were created.
